# Changes in demography and geographic distribution in the weeping pinyon pine (*Pinus pinceana*) during the Pleistocene

**DOI:** 10.1002/ece3.9369

**Published:** 2022-10-05

**Authors:** Laura Figueroa‐Corona, Alejandra Moreno‐Letelier, Diego Ortega‐Del Vecchyo, Pablo Peláez, David S. Gernandt, Luis E. Eguiarte, Jill Wegrzyn, Daniel Piñero

**Affiliations:** ^1^ Posgrado en Ciencias Biológicas Universidad Nacional Autónoma de México Ciudad de México Mexico; ^2^ Departamento de Ecología Evolutiva Instituto de Ecología, Universidad Nacional Autónoma de México Ciudad de México Mexico; ^3^ Jardín Botánico Instituto de Biología, Universidad Nacional Autónoma de México Ciudad de México Mexico; ^4^ Laboratorio Internacional de Investigación sobre el Genoma Humano Universidad Nacional Autónoma de México Juriquilla Mexico; ^5^ Centro de Ciencias Genómicas Universidad Nacional Autónoma de México Cuernavaca Morelos Mexico; ^6^ Departamento de Botánica Instituto de Biología, Universidad Nacional Autónoma de México Ciudad de México Mexico; ^7^ Department of Ecology and Evolutionary Biology University of Connecticut Storrs Connecticut USA

**Keywords:** Chihuahuan Desert, demographic contraction, historical isolation, *Pinus pinceana*, Pleistocene

## Abstract

Climate changes, together with geographical barriers imposed by the Sierra Madre Oriental and the Chihuahuan Desert, have shaped the genetic diversity and spatial distribution of different species in northern Mexico. *Pinus pinceana* Gordon & Glend. tolerates extremely arid conditions. Northern Mexico became more arid during the Quaternary, modifying ecological communities. Here, we try to identify the processes underlying the demographic history of *P. pinceana* and characterize its genetic diversity using 3100 SNPs from genotyping by sequencing 90 adult individuals from 10 natural populations covering the species' entire geographic distribution. We inferred its population history and contrasted possible demographic scenarios of divergence that modeled the genetic diversity present in this restricted pinyon pine; in support, the past distribution was reconstructed using climate from the Last Glacial Maximum (LGM, 22 kya). We inferred that *P. pinceana* diverged into two lineages ~2.49 Ma (95% CI 3.28–1.62), colonizing two regions: the Sierra Madre Oriental (SMO) and the Chihuahuan Desert (ChD). Our results of population genomic analyses reveal the presence of heterozygous SNPs in all populations. In addition, low migration rates across regions are probably related to glacial–interglacial cycles, followed by the gradual aridification of the Chihuahuan Desert during the Holocene.

## Graphical abstract

We focused on how phylogeography, historical demography, and historical migration have influenced the current genetic variation and geographic distribution of P. pinceana, a geographically restricted pinyon pine that tolerates extremely arid conditions in northern Mexico. We analyzed the genomic data of two biogeographic zones where Pinus pinceana, exhibits high differentiation among populations. We used genomic information, along with climatic projections over the Pleistocene and Holocene, to infer the past demographic history of this species as well as its spatial and temporal distribution.

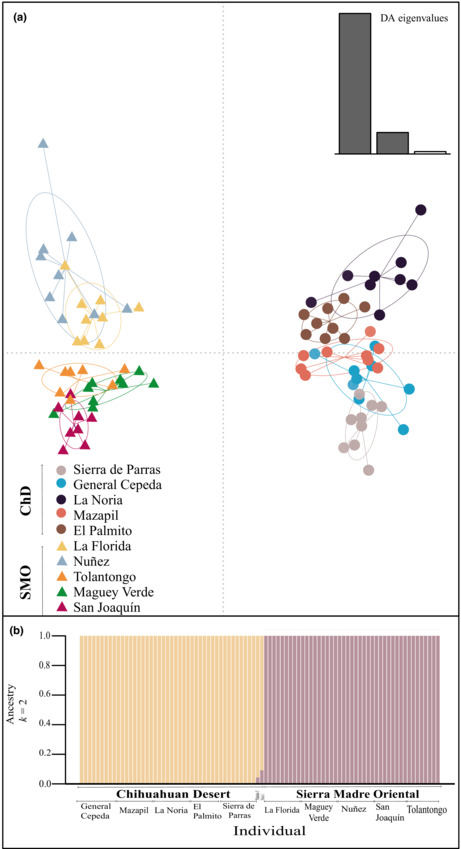

## 1 INTRODUCTION

Chihuahuan Desert (ChD) communities have a long history of high variability in their spatial dimensions, driven primarily by water input, which has fluctuated over time (Noy‐Meir, 1973; Zavala‐Hurtado & Jiménez, 2020).

Geological and climatic events during the Pleistocene affected the diversity patterns of ChD communities (Scheinvar et al., 2020). Several phylogeographic studies have reported dynamic scenarios where populations migrated and expanded to the south through the Central Mexican Plateau during the interglacial (15–20 glacial cycles), followed by events of recolonization of the northern ChD (Van Devender & Burgess, 1985). Such is the case for *Ephedra compacta* (Loera et al., 2017), *Agave lechuguilla* (Scheinvar et al., 2017), and *Pinus remota*, which retreated to Pleistocene refugia in the Bolsón de Mapimí (Lanner & Van Devender, 1981).

The ChD is a high‐elevation desert ranging from 600 to 1675 m a.s.l. and receives more rain than other deserts (235 mm of mean annual precipitation; National Park Service, https://www.nps.gov/im/chdn/ecoregion.htm). The ChD is the third‐largest desert in the American continent, considered the most diverse in the Western Hemisphere and one of the most diverse arid regions in the world (Beck & Gibbens, 1999). It also has both a high species diversity and richness of endemism (Rzedowski & Calderón de Rzedowski, 1993). The ChD landscape maintains different vegetation types, such as grass steppes, xeric shrubs in the intermountain plains and valleys, and open woodlands at higher elevations (Czaja et al., 2014; Van Devender & Burgess, 1985).

The Sierra Madre Oriental (SMO) is a high mountain range (1480–3720 m a.s.l.) dating to the Late Cretaceous and early Tertiary Laramide (80–40 Ma; Eguiluz de Antuñano et al., 2000). With a high climatic diversity due to its complex physiographic heterogeneity and meteorological phenomena (Hernandez‐Cerda & Carrasco‐Anaya, 2004), the SMO receives moisture from the Gulf of Mexico; while the eastern slopes have tropical and temperate forests, the western slope is much drier with xeric scrubs and pine‐oak‐juniper woodlands (Morrone et al., 2017; Rzedowski, 1978).

The ChD biota has experienced climate fluctuations throughout geologic time. Some of these fluctuations occurred during the Late Quaternary. Paleoecological reconstructions suggest that a woodland corridor covered the area between the SMO and the Sierra Madre Occidental in Mexico (Lanner & Van Devender, 1981; Metcalfe, 2006). One of several reductions in pinyon‐juniper and oak woodland occurred during the last 2.5 M years (Pleistocene), which included intense environmental and climatic changes. During the Pleistocene, 11 climatic cycles of growth and reduction in the polar ice cap occurred in North America, affecting the global climatic conditions and consequently the composition of species associated with this woodland corridor. In particular, during the Late Wisconsin glaciation, the vegetation that covered most of the ChD during the Pleistocene was composed of pinyon pines, junipers, and oaks, which started to decrease in extension ca. 11 kya (Holocene), when the reduction in the paleolakes formed during the interglacial period (~115–117 kya) turned this corridor into a warmer and drier area, resulting in a shift to xeric vegetation (Castiglia & Fawcett, 2006; Czaja et al., 2014; Elias & Van Devender, 1990; Lanner & Van Devender, 1981; Ortega‐Ramírez et al., 1998; Ramírez et al., 2003; Van Devender & Burgess, 1985).

*Pinus pinceana* is an endangered conifer, considered a paleorelict (Perry, 1991; SEMARNAT 059, 2010) that inhabits rocky soils with extreme aridity (Passini, 1985). It is locally restricted to the slopes of ChD in the western slopes of the SMO (1480–3000 m a.s.l.; Farjon et al., 1997) with a mean precipitation of 300–400 mm over the summer (Perry, 1991). The dominant trees in these communities are *Pinus cembroides*, *Juniperus* spp., and *Yucca* spp. Morphological differences, like differential needle wax cover, have been reported across the distribution of *P. pinceana* (Martiñón‐Martínez et al., 2010; Ortiz‐Medrano et al., 2016). Previous population genetic studies have shown that *P. pinceana* has a high genetic diversity within and high differentiation among populations (Escalante, 2001; Ledig et al., 2001; Molina‐Freaner et al., 2001).

In this study, we aim to identify how phylogeography, historical demography, and the climatic transformation during the Quaternary influenced the current genetic variation and geographic distribution of *P. pinceana* by addressing the following four goals: (1) characterize its genetic diversity using genome‐wide single nucleotide polymorphisms (SNPs) data, (2) identify phylogeographic patterns across its natural distribution range, (3) infer its past demographic history during the Pleistocene and Holocene, and (4) reconstruct its potential distribution during the LGM to infer the principal climatic limitations of the species.

## 2 METHODS

### 2.1 Sampling and genetic diversity characterization

**FIGURE 1:**
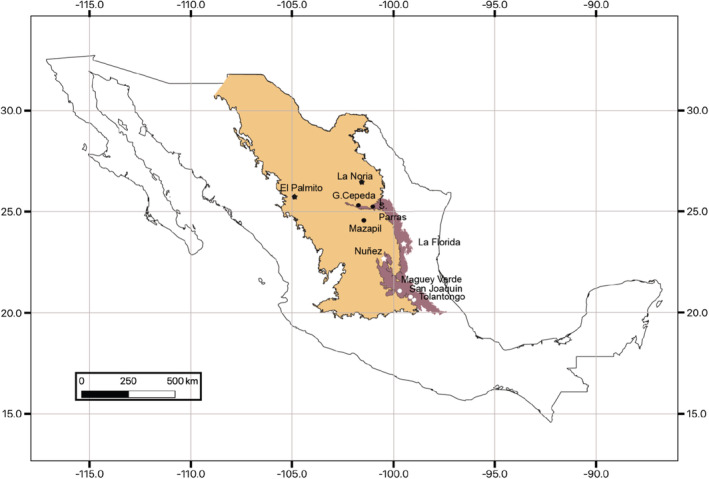
Geographic locations of the 10 populations sampled of *Pinus pinceana* analyzed genetically. The biogeographic province that encompasses the Chihuahuan Desert is located inside the sand color area, and the sampled populations from this province are indicated by a black dot; black stars correspond to Chw. The Sierra Madre Oriental is located inside the purple area, and the sampled populations from this area are shown with white dots; white stars correspond to SMOn.

The 10 sampled localities were distributed across the entire geographic range of *P. pinceana* (Figure S1). Individuals were chosen over the whole area. Usually, these populations are formed by 50–100 trees and we tried to sample the whole ecological area. Needles from nine healthy, regularly distributed adult trees were sampled. Figure 1 shows the regionalization in biogeographic provinces following Morrone et al. (2017).

DNA was extracted using the CTAB protocol (Doyle & Doyle, 1987). Samples with a concentration over 100 ng/ml were double‐digested with the restriction enzymes *Pstl*/*Mspl*, which were tested in silico in the genome of *Pinus lambertiana* to generate fragments along different regions across the genome. The fragments were single‐end sequenced with an Illumina Hi‐Seq 2500 at the Wisconsin Biotechnology Center.

The read adapters were removed using TRIMMOMATIC (v0.36; Bolger et al., 2014). Only sequences with a minimum length of 80 bp and a minimum Phred quality score of 30 were retained. The read quality was visualized with FASTQC (v0.11.7; https://www.bioinformatics.babraham.ac.uk/projects/fastqc/) and MULTIQC (v1.9; Ewels et al., 2016). High‐quality reads were mapped to the *P. lambertiana* genome assembly (Stevens et al., 2016; v1.5) using the BWA‐MEM algorithm (v0.7.10; Li et al., 2009). Unmapped reads were discarded. The MarkDuplicates feature from the Picard package (http://broadinstitute.github.io/picard/) was used to correct artifacts of PCR duplication.

Finally, we used HaplotypeCaller and VariantFiltration tools from the Genome Analysis Toolkit (GATK v3.7; McKenna et al., 2010) to filter SNPs by quality‐by‐depth (QD 2.0), mapping quality (MQ 40.0), Fisher strand bias test (to determine differences in the number of sequences that support the reference and alternate alleles on each strand; FS > 60.0), rank‐sum test for the evaluation of the mapping qualities supporting the reference or alternate alleles (MQRankSum <−12.5), rank sum test for assessment of bias in the position of alleles within the sequences (ReadPosRankSum <−8.0), and strand bias between forward and reverse strands by the symmetric odds ratio test (SOR > 4.0).

We obtained a set of curated SNPs that were filtered prior to analysis using VCFtools (v0.1.16; Danecek et al., 2011). The filtering process removed SNPs with differences compared with the reference but fixed them in *P. pinceana*, followed by keeping biallelic alleles with a maximum of 25% maximum missing data and individuals with <0.2 of missing data, and at least 15× of coverage.

To remove putative paralogs, we used HDplot (v0.5–7; McKinney et al., 2017) to filter polymorphisms with *H* (heterozygosity) larger than 0.6 and *D* (deviation from even read ratios in heterozygous) outside the range of −10 to +10. These parameters were chosen according to the proportion of heterozygous individuals within a population and allelic ratios within heterozygous individuals. A filter based on linkage disequilibrium (LD) was applied using PLINK v1.9 (with a window size of 50 bp, LD < 0.5, and window shift of 5; Purcell et al., 2007). An additional filter was applied to eliminate polymorphisms out of Hardy–Weinberg equilibrium (Figure S2). Based on these filtering parameters we expected to get a better representation of neutral loci, reducing the artifact incorporated by the abundance in paralogs and pseudogenes, which are abundant in conifers (Prunier, Verta, & MacKay, 2016), and giving more confidence in the accuracy of variant calls to the coalescent models. We retained a total of 3100 SNPs in 88 individuals.

We estimated the genetic diversity for the 10 populations using observed heterozygosity (*H*_O_), within‐population gene diversity (*H*_*S*_), and the inbreeding coefficient (*F*_IS_) using the *R* package *Hierfstat* (Goudet, 2005). Additionally, DNAsp (v6; Rozas et al., 2017) was used to calculate allelic richness, per site nucleotide diversity (*π*), and pairwise population differentiation (*F*_ST_; Weir & Cockerham, 1984).

### 2.2 Phylogeographic and population structure

To estimate the clustering among samples without any prior geographic provenance, we ran a discriminant analysis of principal components (DAPC) with the *r* package *Adegenet* (Jombart & Ahmed, 2011). Then, we conducted an ADMIXTURE analysis (v1.3.0; Alexander & Lange, 2011) using the genotypes that were previously converted to ordinary PLINK files (.ped) using PLINK (v1.9; Purcell et al., 2007) to estimate the population structure among individuals for *K* values from 2 to 10 admixture proportions. We selected the replicate with the lowest log‐likelihood value for each value of *K*. Then, we chose the value of *K* with the lowest cross‐validation error.

### 2.3 Hypotheses of demographic history

The demographic history of *P. pinceana* was inferred by testing four different scenarios via the composite likelihood approach of fastsimcoal (v2.6; Excoffier & Foll, 2011; Excoffier et al., 2013), using the site (allele) frequency spectrum (SFS) of 3100 SNPs filtered without bias of frequency computed by *easySFS.py* (Figure S4; https://github.com/isaacovercast/easySFS), to infer the divergence times between and within biogeographic regions (Figure 1).

**FIGURE 2:**
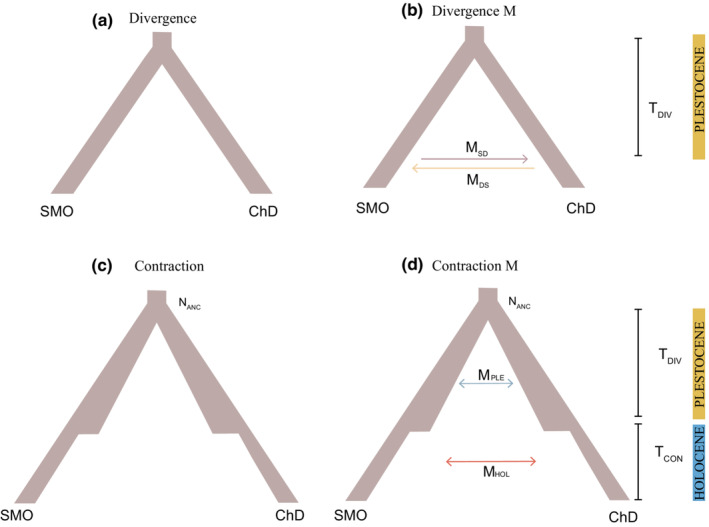
Demographic histories modeled for *Pinus pinceana*. (a) Divergence, the model draw for the divergence between SMO and ChD. (b) Divergence M includes asymmetrical and bidirectional migration among regions.

Following the clustering of DAPC, our four scenarios modeled included two scenarios without migration: Divergence, to estimate divergence times between ChD and SMO populations (*T*_DIV_), and effective population sizes (*N*_*e*_). Contraction, to estimate the divergence times between the ChD (*T*_DIV_) and SMO populations, followed by the independent contraction of both groups during the Holocene. We also modeled two scenarios M, including migrations, based on the former two scenarios with the same parameter ranges but adding independent migration rates among groups (Figure 2).

For each scenario, we estimated divergence times converted from generations to years using a generation time of 40 years. This generation time represents an approximation of the average age of reproductive individuals in a population. It is based on seed production and the proportion of viable seeds through the lifespan in another pinyon pines species (*Pinus nelsonii*) related to *Pinus pinceana* (Suzan‐Azpiri, Sanchez‐Ramos, Martínez‐Avalos, Villa‐Melgarejo, & Franco, 2002). The mutation rate was set at 7.28 × 10^−10^ per base pair per year, as reported for Pinaceae (i.e., 2.91 × 10^−8^ per base per generation; De la Torre et al., 2017).

The Divergence without migration model was set using search ranges for the effective population sizes (*N*_*e*_) from 100 to 1000 individuals in the SMO and ChD lineages, and *T*_DIV_ between 275 and 62,500 generations ago. The Contraction model was set using search ranges for the effective population sizes (*N*_*e*_) of SMO and ChD from 100 to 1000 individuals. *T*_DIV_ was set to date the divergence event within ChD and SMO between 275 and 62,500 generations ago, and *T*_CON SMO_ was set to date a contraction event in the SMO group and *T*_CON ChD_ using the range of 40 and 275 generations ago. Finally, for the two migration models, we used the same range adding between 1 × 10^−9^ and 1 × 10^−4^, per base per generation, drawn from a log uniform prior distribution.

For each of the four models, we simulated 100 replicates, 150,000 iterations, and 30 ECM cycles. To select the best scenario, we estimated the Akaike Information Criterion AIC value (Akaike, 1974) using the maximum composite likelihood estimated from the best replicate and compared the models using the weighted Akaike information criterion (wAIC). Finally, we calculated confidence intervals of parameter estimates from 100 parametric bootstrap replicates by simulating SFS from the maximum composite likelihood estimates and re‐estimating the parameters each time (Excoffier et al., 2013).

### 2.4 Ecological niche modeling and refugia hypotheses

We compiled 37 records verified from Escalante (2001), Figueroa‐Corona (2012) and Figueroa‐Corona, Delgado, Wegrzyn, & Piñero (2021) and specimen records from the National Herbarium of Mexico (MEXU; Figure S1). Each presence point was separated by at least 1 km from other presence points, to avoid spatial autocorrelation. We extracted the values for 19 bioclimatic variables with a resolution of 30 arc‐seconds from CHELSEA (Krager et al., 2017) using QGIS (v3.10; https://qgis.org/es/site/) and performed an outlier analysis using Mahalanobis distances using the package JMP (v9; SAS Institute Inc., 2010).

To reduce model overfitting, we selected independent bioclimatic variables before constructing the ENMs with a Principal Component Analysis using JMP (v9; SAS Institute Inc., 2010). We retained the variables that had the higher loading values for the first three principal components (>0.7) and had a correlation coefficient of <0.8: Annual Mean Temperature (Bio1), Isothermality (Bio3), Temperature Seasonality (Bio4), Annual Precipitation (Bio12), and Precipitation in the Driest Month (Bio14) from PC1; Precipitation Seasonality (Bio15), and Precipitation of Warmest Quarter (Bio18) from PC2; and Minimum Temperature of Coldest Month (Bio6) from PC3.

To construct a potential niche model, we used Maxent (v3.3.3e; Phillips & Dudík, 2008) implemented in the suite Wallace (Kass et al., 2018; Appendix S1), testing linear, quadratic, product, and hinge features and regularization multipliers from 0.5 to 2 with 0.5 intervals. The best fitting model was selected with the ΔAIC (ENMeval implemented by Wallace). The details of the analyses can be found in the R script available in the Appendix S1. We processed the selected model (LQHP Rm = 1.5) by removing the 10th percentile training probability, as described by Liu et al. (2005).

The projection to the Last Glacial Maximum bioclimatic layers from CHELSEA on the PMIP3 data (Krager et al., 2021) was performed using the same parameters for the current distribution. Areas for each model (current and LGM) were estimated by counting pixels with the package *raster* (Hijmans & van Etten, 2012), where each pixel corresponds to approximately 1 km^2^.

## 3 RESULTS

### 3.1 Genotyping and genetic characterization

The single‐end sequences produced an average of 2.8 M reads per individual, which was reduced to 2.54 M reads after quality control. The datasets obtained with GATK produced 605,489 variants. VCFtools was used to obtain the final processed data with 3100 biallelic SNPs, with a moderate amount of missing data by allele (mean = 0.0912%), and a mean depth per site of 64×.

Globally, the observed heterozygosity (*H*_O_) was 0.0663, the within‐population gene diversity (*H*_S_) was 0.0541, and the inbreeding coefficient (*F*_IS_) was −0.2247 (Table 1). Levels of within‐population gene diversity (*H*_*S*_) were similar among populations and ranged from 0.0388 (Maguey Verde, SMO) to 0.0686 (General Cepeda; ChD). Levels of *H*_O_ were highest for the ChD location, General Cepeda (0.0824), and the lowest for Maguey Verde (0.0463) in SMO. Estimates for the inbreeding coefficient *F*_IS_ were negative for all populations, ranging from −0.0874 (Maguey Verde; SMO) to −0.1439 (Sierra de Parras; ChD), while the highest nucleotide diversity was found in Sierra de Parras (0.002), and the lowest in La Florida (0.0004).

**TABLE 1:** Measures of genetic diversity for 88 individuals of *P. pinceana* from ten populations calculated from 3100 single nucleotide polymorphism loci

Population		*H*_S_	*H*_o_	*F*_IS_	*π*	Tajima D	*θ*_W_
SMO	Tolantongo	0.049	0.058	−0.0881	0.0009	−0.6791_NS_	0.0811
	San Joaquín	0.0453	0.055	−0.1117	0.0011	−0.5995_NS_	0.0767
	Maguey Verde	0.0388	0.0463	−0.0874	0.0009	−0.7144_NS_	0.0611
	Nuñez	0.0512	0.0636	−0.1158	0.0012	−0.6266_NS_	0.08
	La Florida	0.0469	0.0569	−0.1073	0.0004	−0.6251_NS_	0.0748
ChD	El Palmito	0.0494	0.0607	−0.1023	0.0011	−0.6596_NS_	0.0744
	Mazapil	0.0612	0.0748	−0.1201	0.001	−0.5852_NS_	0.0892
	La Noria	0.0638	0.0792	−0.1328	0.0017	−0.5478_NS_	0.0919
	G. Cepeda	0.068	0.0848	−0.139	0.0015	−0.4724_NS_	0.0943
	Sierra Parras	0.0654	0.0824	−0.1439	0.002	−0.4688_NS_	0.096
Global		0.0541	0.0663	−0.2247	0.0006	−0.7457_NS_	0.147

Abbreviations: *H*_O_, observed heterozygosity; *H*_E_, expected heterozygosity within populations; *F*_IS_, inbreeding coefficient; *π*, nucleotide diversity.

### 3.2 Genetic structure and phylogeography pattern

The global *F*_ST_ value was 0.097 (Table 2). Within SMO, the populations had a mean *F*_ST_ = 0.0144, compared to 0.012 within the ChD populations. Between biogeographic regions, the mean value was 0.0536.

**TABLE 2:** Pairwise differentiation (*F*_ST_) among the 10 populations sampled of *P. pinceana* with 3100 SNPs

	Tolantongo									
SMO	San Joaquín	0.0078								
	Maguey Verde	0.0099	0.01							
	Nuñez	0.01	0.0185	0.02						
	La Florida	0.0121	0.021	0.0214	0.0128					
ChD	El Palmito	0.0439	0.0471	0.0445	0.0473	0.0453				
	Mazapil	0.0469	0.0494	0.0526	0.0546	0.0536	0.0162			
	La Noria	0.0482	0.051	0.0541	0.0532	0.0525	0.0124	0.01		
	G. Cepeda	0.0536	0.0575	0.0596	0.061	0.0589	0.0184	0.008	0.008	
	Sierra Parras	0.0574	0.0577	0.0626	0.065	0.0626	0.0219	0.008	0.013	0.005

**FIGURE 3:**
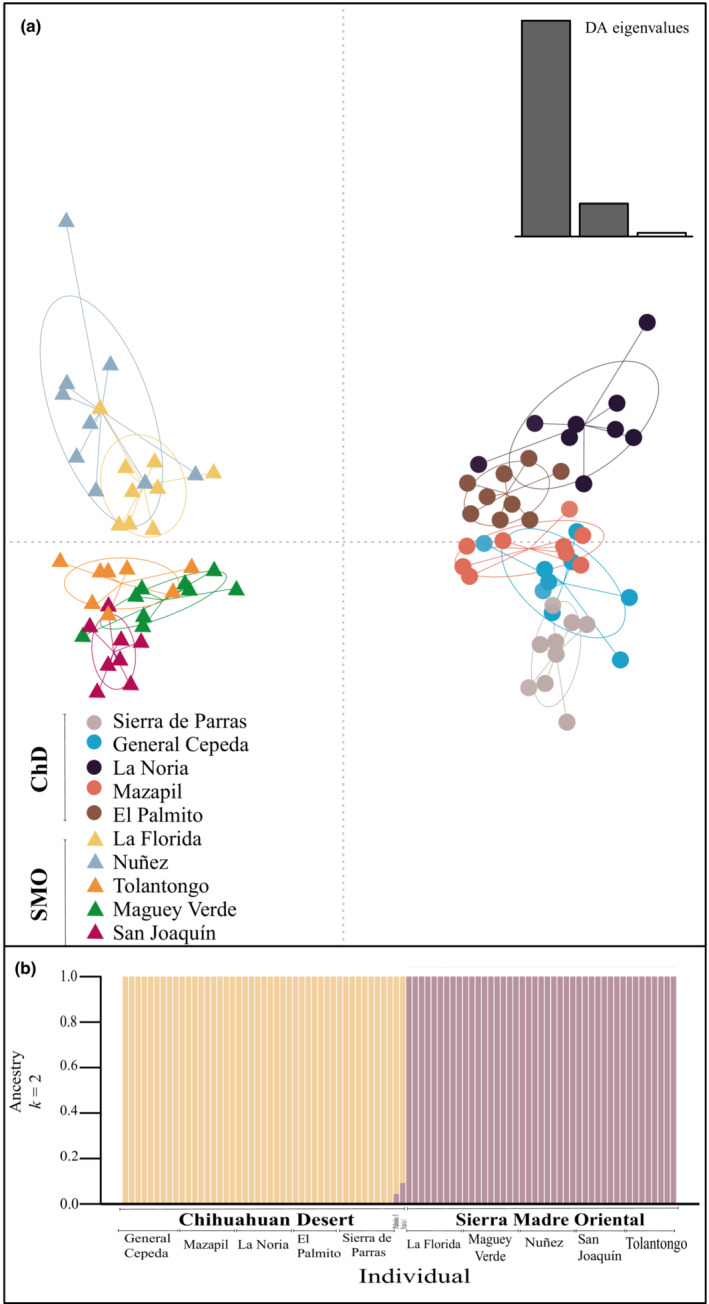
Population structure and nucleotide diversity based on 3100 genome‐wide SNPs in *Pinus pinceana*. (a) Scatterplots resulting from discriminant analysis of principal components (DAPC) for 88 samples, and the eigenvalues of discriminant functions portrayed in the bottom right. (b) Admixture proportions of all the individuals for ancestral populations (*k*) for *K* = 2. Each bar represents one individual and shows its admixture proportions.

The discriminant analysis of principal components (DAPC) showed two clear population clusters divided by PC1, describing 17.14% of the total variance while PC2 describes 4.3% of the total variance. PC2 helps to divide the SMO cluster with one subcluster including Tolantongo, San Joaquín, and Maguey Verde and another including La Florida and Nuñez (Figure 3a).

The ADMIXTURE analysis showed that *K* = 2 supports the clustering in two biogeographic areas. For *K* = 2, 42 of the 45 samples from SMO were classified 100% in one cluster (Figure 3b). Forty‐three of the 45 samples from ChD were classified in the first cluster, while two samples from the northwest population of El Palmito were found with a level of admixture between both groups. The higher values of *K* tested (3–9) showed sub fragmentation within these two biogeographic areas but with less likelihood.

### 3.3 Demographic history

We used the entire 3100 SNP dataset to model the population divergence time hypotheses from the demographic simulations. Three of the four scenarios resulted in a divergence time estimated during the Early Pleistocene while the Contraction M scenario estimated the divergence between clusters during the Miocene (Tables S1 and S2).

**FIGURE 4:**
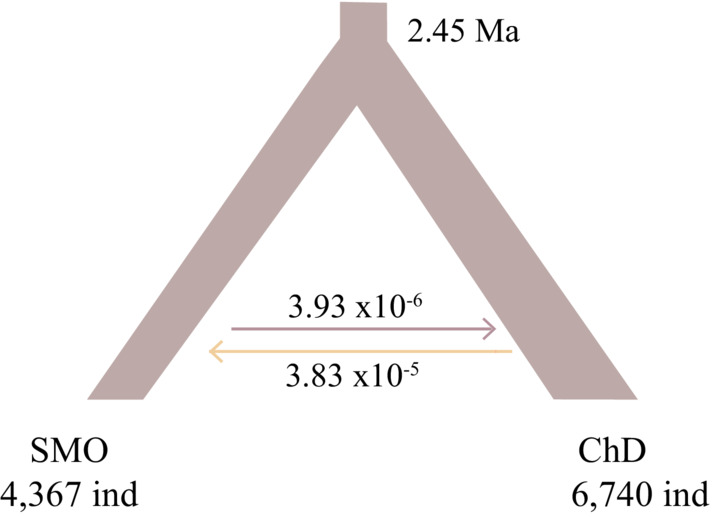
Divergence with migration hypothesis selected as the best‐fit model along comparisons with resulting values for each parameter. Parameter values for this and additional models are provided in Tables S1 and S2.

The best‐supported model was the divergence with migration according to the ΔAIC that considers the split into two lineages according to the biogeographical regions (Figure 4; Table S3; Appendix S1). The dated divergence (*T*_DIV_) between them was estimated in the Early Pleistocene (2.49 Ma; 95% CI: 3.28–1.62). Effective population sizes (*N*e) for groups were similar between groups, SMO with 4367 individuals (95% CI: 4958.59–3775.41), and ChD with 6740 individuals (95% CI: 7531.42–5948.58). Migration rates per generation (*m*) were estimated to be significantly lower 3.93 × 10^−6^ (95% CI: 1.364 × 10^−5^–5.78 × 10^−6^) from SMO to ChD, than in the opposite direction (3 × 10^−5^; 95% CI: 3.86 × 10^−5^–3.8 × 10^−5^).

### 3.4 Historical distribution projections

**FIGURE 5:**
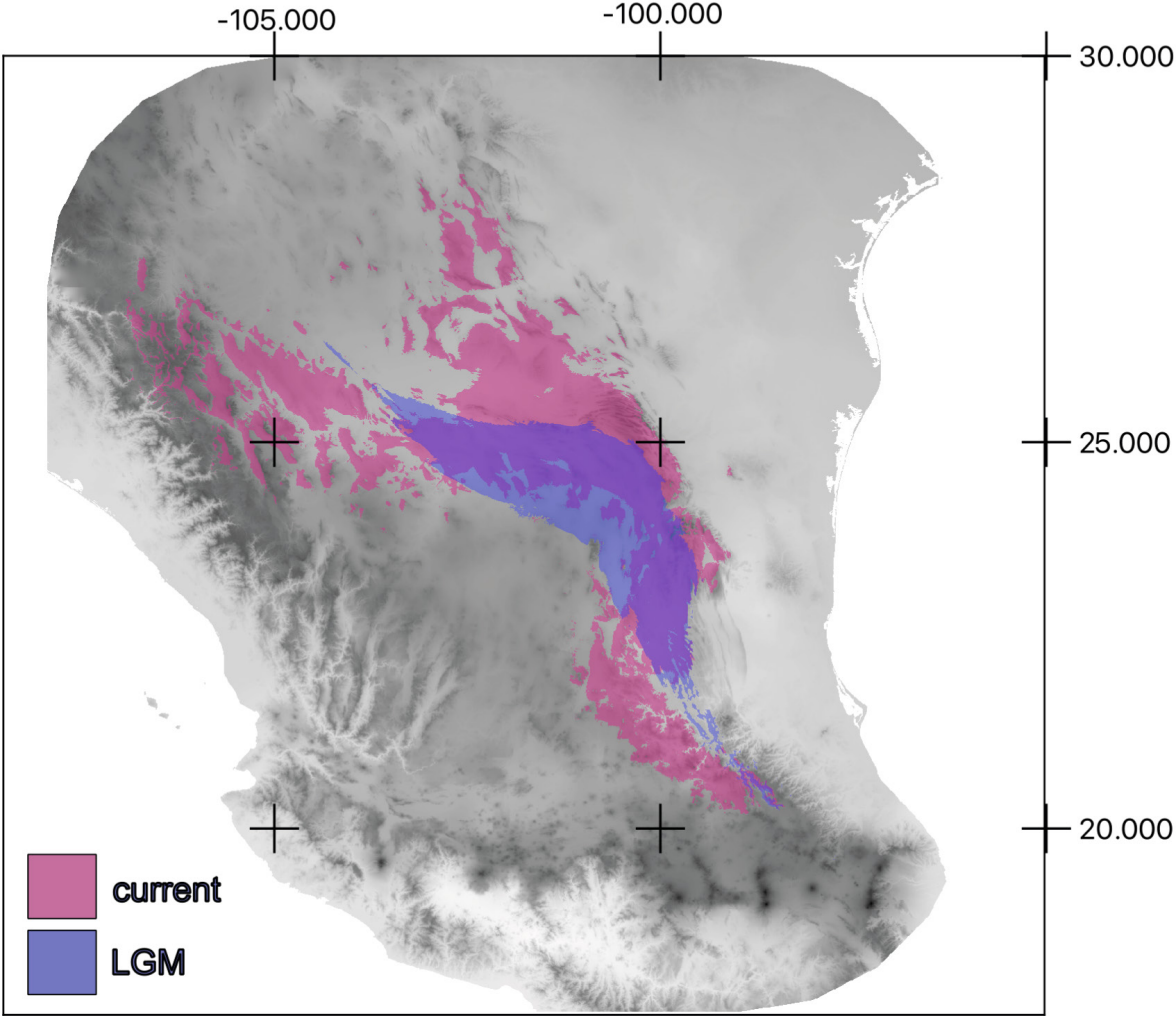
Ecological niche modeling predictions of *Pinus pinceana* current climate change during the last interglacial (LIG), and present.

The LGM model showed a reduced suitable area compared with the current model (Figure 5), centered in the northern part of the Sierra Madre Oriental and the Northern Plateau. The LGM covered an area 56% smaller than the current distribution (80,445 pixels in LGM vs. 182,294 pixels for current prediction). There was no suitable area predicted for the Sierra Madre Occidental foothills and the southern part of the Sierra Madre Oriental. Flatlands and basins seem to be a barrier to the distribution of *P. pinceana*, as the areas identified as current or past water bodies have not been occupied by the species. The areas that remained stable, that is, with suitable conditions for *P. pinceana* since the LGM include the current populations of General Cepeda and Mazapil, covering an area of 55,582 pixels (1 pixel is around 1 km^2^).

## 4 DISCUSSION

### 4.1 Phylogeography

We detected a clear geographic structure of genetic diversity of *P. pinceana* between the Chihuahuan Desert and the Sierra Madre Oriental. A similar differentiation has been described in several desert scrub plant species in this area, as we discuss below. This structure is apparently related to climate dynamics during the Quaternary (Castiglia & Fawcett, 2006; Czaja et al., 2014; Ramírez et al., 2003). These changes highlight the importance of two distinct processes in the Chihuahuan Desert: first, genetic drift due to reductions in effective population sizes in different areas; and second, isolation of populations due to low migration rates.

The genetic diversity (*H*_O_) patterns using SNPs were similar to the values described in conifer species with broad geographic ranges in Mexico, like *Pinus patula*, *Abies religiosa*, and *Juniperus blancoi* (Giles‐Pérez et al., 2022; Peláez et al., 2020; Reyes, 2016). By contrast, the genetic diversity values previously reported for microsatellite (cpDNA) SSR loci in *P. pinceana* (Escalante, 2001; Figueroa‐Corona, 2012) were substantially higher than the values found here. This is expected because microsatellites have higher mutation rates (Hamblin et al., 2007).

In accordance with previous reports in *P. pinceana* (Ledig et al., 1986, 2001; Molina‐Freaner et al., 2001), we found a high presence of heterozygous alleles and consequently negative *F*_IS_ within populations. Similar situations with high heterozygosities and in some cases negative *F*_IS_ averages have been found in some conifers like *Cryptomeria japonica* var. *sinensis*, and *Pinus patula* (Cai et al., 2020; Peláez et al., 2020), while in *P. albicaulis*, the estimates have been positive (Liu et al., 2016).

We attribute these negative *F*_IS_ values to two possibilities, first the presence of paralogous SNPs that were not filtered with the approach of McKinney et al. (2017), as has been suggested in view of the complexities of conifer genomes (De la Torre et al., 2014; Leitch & Leitch 2012). The second possibility is the existence of natural selection as described in *P. patula* by Peláez et al. (2020). Disentangling these possibilities would require a different experimental design to analyze parental heritance or survival of seedings, in addition to better recognition of the organization of the *Pinus pinceana* genome.

The differentiation of the SMO and ChD was attributed to isolation by distance in a previous study based on cpDNA SSR loci (Escalante, 2001). Our results of genetic structure clearly identified two lineages, corresponding to the same two biogeographic regions. Furthermore, these lineages present relatively high levels of genetic differentiation between them (*F*_ST_ = 0.097), like that typically reported for conifer taxa with wide distribution ranges (Cobo‐Simón et al., 2020; Jaramillo‐Correa et al., 2020; Peláez et al., 2020). Nevertheless, the discriminant components analysis shows that SMO and ChD groups have significant levels of admixture between them.

The structured phylogeographic pattern between the ChD and SMO that we detected has been reported in several desert scrub plant species from the ChD (e.g., Sosa et al., 2009; Scheinvar et al., 2017; Vásquez‐Cruz & Sosa, 2016). In all cases, a strong phylogeographic structure found within the SMO was correlated with geologic and paleoclimatic changes. As an example of this pattern, Vásquez‐Cruz & Sosa (2016) identified a main phylogeographic pattern in species of Rosaceae where the ChD was the ancestral area, followed by contraction of suitable habitat during the Last Interglacial (~120,000–140,000 years), then an expansion during the LGM (~22,000 years) to the SMO and the Central Mexican Plateau and finally recolonization of the ChD during the Mid‐Holocene (~6000 years). Loera et al. (2017) argued that this structured pattern between SMO and ChD in *Ephedra compacta* was reinforced by the lack of dispersal and changes in elevation due to biogeographic barriers within this region.

### 4.2 Demographic history

With a patchy geographic distribution, *P. pinceana* is not considered an endangered species by the IUCN, but it is considered endangered by Mexican law based on more detailed ecological, geographic, and genetic information (SEMARNAT 059, 2010). Demographic data can help us to apply historical context to this question. The current genetic differentiation patterns of the ChD of the xeric scrubs *Ephedra compacta* and *Agave lechuguilla* (Loera et al., 2017; Scheinvar et al., 2017) can be explained by an initial expansion, followed by processes of isolation between lineages. In the case of *P. pinceana* the isolation is related to the present very reduced zones of secondary contact, and limited migration evidencing the biotic transformation during the Quaternary (Elias & Van Devender, 1990;Lanner & Van Devender, 1981; Van Devender & Burgess, 1985).

The results of the four scenarios that we analyzed give support consistent with changes in climate and the genetic structure found in *P. pinceana* in the ChD during the Middle Pleistocene. We suggest that this allopatric fragmentation of two lineages, together with an expansion of the xeric scrubs and pine‐oak forests after the end of the glacial inter‐cycles, was crucial in shaping the present distribution of the genetic diversity in *P. pinceana*.

Quaternary dynamics in the ChD have been described with geological and biogeographic evidence in the xeric scrub and nonarboreal species in the region. These results agree with the pattern of the geographic distribution of genetic diversity found in this study. In particular, for *Agave lechugilla*, Scheinvar et al. (2020) described contrasting levels of genetic diversity between the northern and southern parts of the ChD. On the other hand, genetic structure and demographic history during the Pleistocene were found in different species with a diversity of life history traits, reproductive strategies, and demographic dynamics, for example, in xeric scrubs *Agave lechuguilla*, *A. striata*, and *A. stricta* (Martínez‐Ainsworth, 2013; Scheinvar et al., 2017; Trejo et al., 2016), the rodents *Thomomys umbrinus* and *Perognathus avus*, and the turtle *Kinosternon avescens* (Mathis et al., 2014; Neiswenter & Riddle, 2010; Serb et al., 2001). The estimated migration rates in our study show an asymmetric flux between regions (Table S2). Nevertheless, we still cannot establish a cause for the percentage of admixture in individuals from El Palmito given the low connectivity with nearby populations that was suggested by the ADMIXTURE analysis (Figure 3).

### 4.3 Genetic diversity in a temporal context

Phylogenetic divergence within *Pinus* subsection *Cembroides* is estimated to have occurred during the Miocene (11 Mya; Gernandt et al., 2008; Saladin et al., 2017) or the Oligocene (Jin et al., 2021). The fossil evidence for pinyon pines records large changes during the Pliocene in their distribution throughout the Central Mexican Plateau (Lanner & Van Devender, 1981; Van Devender & Burgess, 1985). Conifers had a wider distribution during the Pleistocene in Mexico in the ChD, and a complex dynamic around the Holocene that diminished or extremely reduced their populations (Jaramillo‐Correa et al., 2006; Quiñones‐Pérez et al., 2017).

Morpho‐physiological studies in *P. pinceana* detected morphological differentiation patterns, where the individuals from the ChD had thicker epicuticular waxes on the needles, faster growth, larger primary roots, and greater volume of secondary roots in Chihuahuan Desert individuals than in individuals from the Sierra Madre Oriental, differences that suggest local morphological adaptation to aridity exposure (Córdoba‐Rodríguez et al., 2011; Martiñón‐Martínez et al., 2010). We could not ascribe any adaptive importance to the differentiation of the genetic clusters, probably because of the absence of candidate genes adapted to climatic factors across regions. An independent analysis using comparative transcriptomics for this species in both biogeographic regions detected a possible plastic response to biotic and abiotic factors present in the populations (Figueroa‐Corona et al., 2021).

### 4.4 Historical distribution

Based on our analyses, we propose that *P. pinceana* differentiated into two lineages in the Middle Pleistocene (~2.49 Ma; 95% CI: 3.28–1.62), followed by the division of both biogeographic regions (~127.7–~539.2 kya) during the interglacial cycles. The climatic conditions in the habitat of *P. pinceana* are more suitable at present than they were during the LGM; this is in contrast to what has been described in other conifers like *Picea chihuahuana*, which has become more restricted due to the disappearance of a suitable habitat making it susceptible to the loss of genetic variability via genetic drift, inbreeding depression and strong selection (Jaramillo‐Correa et al., 2006; Quiñonez‐Perez et al., 2017).

The recent climatic changes in the ChD, the increment in aridification, and the reduction in water bodies (Czaja et al., 2014) promoted the geographic expansion of *P. pinceana*, and the phylogeographic patterns inferred from our data, involving a gradual expansion over the ChD since the LGM, in particular to the southern and western part of the distribution. This pattern of expansion has been described in the ChD in other scrub plant species like *A. lechuguilla* and *E. compacta* (Loera et al., 2012; Scheinvar et al., 2017). The lack of compatible environmental layers to other points in the past prevents us from exploring the distribution closer to the divergence of the two lineages.

Mazapil and General Cepeda are two of the three most genetically diverse populations but also represent, according to the estimation of the habitat, the most stable region over the climate dynamics since the interglacial cycles. Thus we interpreted that this region was a refuge for *P. pinceana*. Indeed this region has not been previously described as a refuge for the ChD (Gámez et al., 2017; Loera et al., 2017; Scheinvar et al., 2017, 2020; Vásquez‐Cruz & Sosa, 2020).

## 5 CONCLUSIONS

The genetic diversity in *P. pinceana* was modified by dynamics during interglacial cycles in the Pleistocene. The demographic scenarios studied resulted in a ranking of models that were useful in gauging relative support for competing hypotheses.

In particular, the best model involved the divergence into two lineages in the Middle Pleistocene (~2.49 Ma; 95% CI: 3.28–1.62). These lineages later colonized two biogeographic regions, the SMO and the ChD, while interglacial cycles modified the Chihuahuan Desert as the aridification increased and paleolakes shrank. This division probably occurred during the climatic changes of the Pleistocene and was related to the glacial–interglacial cycles.

Thus, the phylogeographic history of *P. pinceana* is likely explained by climate dynamics that left perceptible marks in the patterns of genetic diversity and structure observed in the species today.

## AUTHOR CONTRIBUTIONS

**Laura Figueroa‐Corona:** Data curation (lead); formal analysis (lead); investigation (lead); methodology (equal); resources (equal); software (equal); visualization (equal); writing – original draft (equal); writing – review and editing (equal). **Alejandra Moreno‐Letelier:** Formal analysis (equal); methodology (equal); visualization (equal); writing – review and editing (equal). **Ortega‐Del Vecchyo Diego:** Data curation (equal); formal analysis (equal); methodology (equal). **Pablo Pelaez:** Data curation (equal); formal analysis (equal); software (equal). **David S. Gernandt:** Formal analysis (equal); funding acquisition (equal); investigation (equal); writing – original draft (equal); writing – review and editing (equal). **Luis E. Eguiarte:** Formal analysis (equal); investigation (equal); supervision (equal); writing – review and editing (equal). **Jill L. Wegrzyn:** Formal analysis (equal); methodology (equal); software (equal); writing – review and editing (equal). **Daniel Piñero:** Conceptualization (equal); data curation (equal); formal analysis (supporting); funding acquisition (lead); investigation (equal); project administration (lead); resources (equal); supervision (lead); writing – original draft (lead); writing – review and editing (lead).

## CONFLICT OF INTEREST

All authors contributed to the manuscript writing and revisions, and declared no conflict of interest.

### OPEN RESEARCH BADGES

This article has earned Open Data and Open Materials badges. Data and materials are available at https://www.ncbi.nlm.nih.gov/bioproject/PRJNA719106/.

## Supporting information


Appendix S1

## Data Availability

Raw reads are available in the SRA NCBI database (https://www.ncbi.nlm.nih.gov/sra/) deposited under the PRJNA719106 BioProject.
