# Supplementary material for: Changes in demography and geographic distribution in the weeping pinyon pine (Pinus pinceana) during the Pleistocene
Source: Ecol Evol. 2022 Oct 5;12(10):e9369. doi: 10.1002/ece3.9369 (PMC9534753; doi:10.1002/ece3.9369)
Supplement: Supplementary file 1 — Appendix S1 [file ECE3-12-e9369-s001.docx]

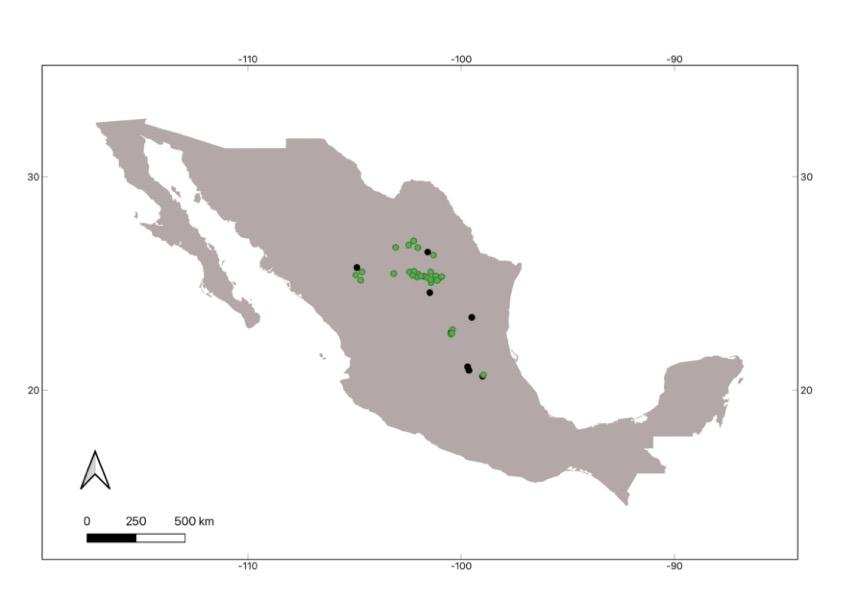


**Figure S1.** *Pinus pinceana* locations processed. Black dots correspond to locations genetically analyzed and used for ecological niche modeling analysis. Green dots represent locations processed only for ecological niche modeling analysis*.*

*
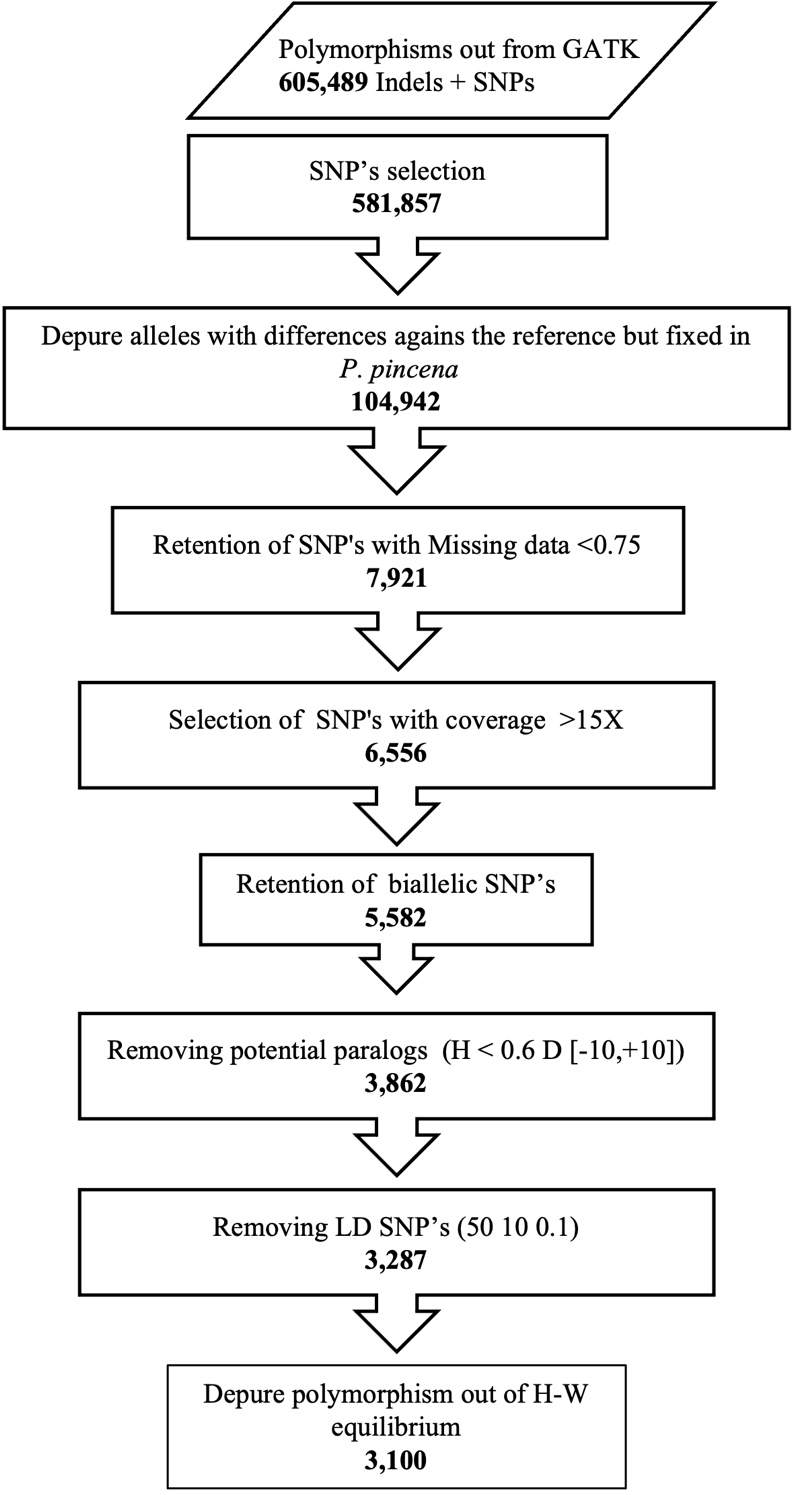
*

# Figure S2. Processing flowchart of SNP’s curation.

#
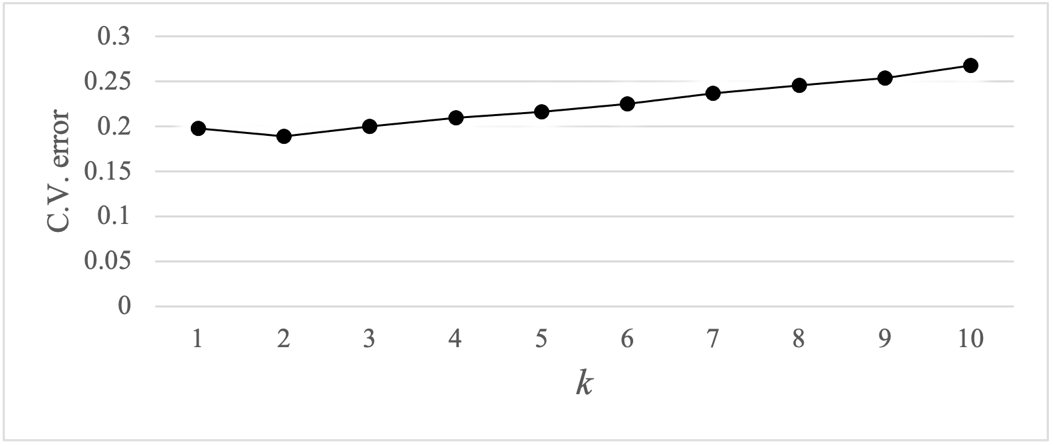


# Figure S3. The cross validations average error across 10 replicates inferred with ADMIXTURE for *k*= 1 to *k*= 10.

*
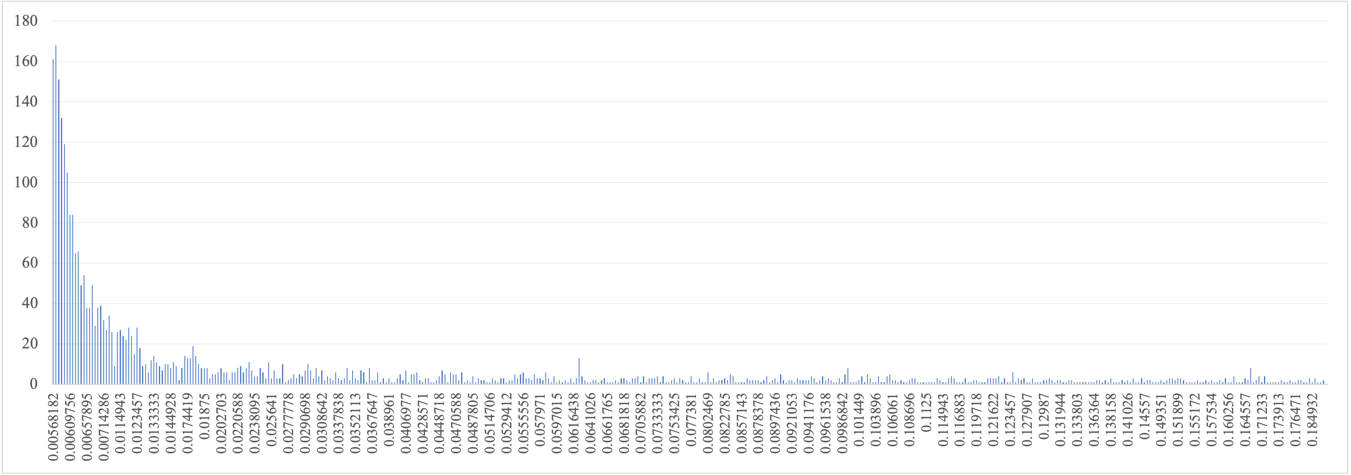
*

**Figure S4.** Site frequency spectrum for 3100 SNP’s used on the demographic analyses

**Supplementary Tables**

| **Table S1.** Parameters inferred for time divergence and effective population sizes from coalescent simulations with fastsimcoal under the demographic models | | | | | | | |
| --- | --- | --- | --- | --- | --- | --- | --- |
|  | ***Ne*** | | | | ***T_DIV_*** | | |
|  | ***SMO*** | ***ChD*** | | |  |  |  |
| **Divergence** | 6,468 | 6,648 | | | 2,446,920 | | |
| **Divergence M** | 4,367 | 6,740 | | | 2,456,960 | | |
|  | ***Ne*** | | | ***T*** | | | |
|  | ***SMO*** | ***ChD*** | ***Ancestral size*** | ***_DIV_*** | | ***_CON SMO_*** | ***_CON ChD_*** |
| **Contraction** | 1,132 | 2,391 | 2,994 | 2,472,400 | | 6,240 | 4,000 |
| **Contraction M** | 666 | 1,439 | 2,784 | 8,030,280 | | 3,160 | 2,760 |
| Population names refers to the *N_e_* number of individuals in the different groups; T_DIV,,_ T _CON SMO_ and T _CON_ _ChD_ are in years; SMO: Sierra Madre Oriental, SMO: Tolantongo, San Joaquín, Maguey Verde Nuñez and La Florida populations, ChD: Chihuahuan Desert, Mazapil, General Cepeda, Sierra de Parras, El Palmito and La Noria populations. | | | | | | | |

| **Table S2.** Migration rates inferred from coalescent simulations with fastsimcoal under the demographic models | | | | |
| --- | --- | --- | --- | --- |
| **Divergence M** | |  | SMO | ChD |
|  |  | SMO |  | 3.93x10^-6^ |
|  |  | ChD | 3.83x10^-5^ |  |
| **Contraction M** | *M_Ple_* |  | ChD | |
|  |  | SMO | 1.62x10^-6^ | |
|  | *M_Hol_* |  | ChD | |
|  |  | SMO | 8.84x10^-6^ | |

| **Table S3.** Comparison of demographic models analyzed with fastsimcoal | | |  |
| --- | --- | --- | --- |
| **Model** | **AIC** | **ΔAIC** | |
| Divergence | 37,335.2378 | 2108.7737 | |
| Divergence M | 35,226.464 | 0 | |
| Expansion | 38,487.6719 | 3261.2078 | |
| Expansion M | 36683.7373 | 1457.2733 | |
